# 493. Incidence and Characterization of Chronic Active COVID Among Patients Infected with the Novel Coronavirus (COVID-19) Receiving B-cell Depleting Therapies (BCDTs)

**DOI:** 10.1093/ofid/ofab466.692

**Published:** 2021-12-04

**Authors:** Jacob Kartes, Emily S Spivak, Hannah Imlay

**Affiliations:** University of Utah, Salt Lake City, Utah

## Abstract

**Background:**

There have been reports of COVID-19 infection characterized by prolonged viral replication [chronic active COVID-19 (CAC)] among immunocompromised patients, including those receiving B-cell depleting therapies (BCDTs). We aimed to characterize the severity and incidence of CAC among patients on BCDTs with COVID-19, and to identify associated risk factors.

**Methods:**

We retrospectively reviewed all patients who received an anti-CD20 BCDT within 1 year of a positive COVID test at University of Utah Health. Demographics, comorbidities, indications, and timing of BCDT were documented. Chart review was performed to characterize the clinical course, including need for hospitalization, COVID-specific therapies, need for ICU and ventilatory support, and mortality. We defined CAC as: (1) despite initial clinical improvement, progression of illness extending beyond 14 days, characterized by ongoing fevers or progressive respiratory failure; or (2) ongoing symptoms with demonstration of absent seroconversion ≥ 14 days into illness. In some patients the diagnosis of CAC was supported by low viral PCR crossing thresholds that occurred ≥ 14 days into illness. Logistic models were used to identify risk factors for CAC among the cohort of patients who survived through the initial period of infection.

**Results:**

We identified 66 individuals who received a BCDT within 1 year of a positive COVID test; 29 (44%) were hospitalized, 4 (6%) required ventilation, and 7 (11%) died within 60 days. Among 63 patients who survived their initial COVID course, 16 (25%) had courses compatible with CAC. Nine (56%) who received a BCDT within 1 month before or 2 weeks after their COVID diagnosis developed CAC; OR 7.4 (95% CI 1.7, 31.6, p=0.002).

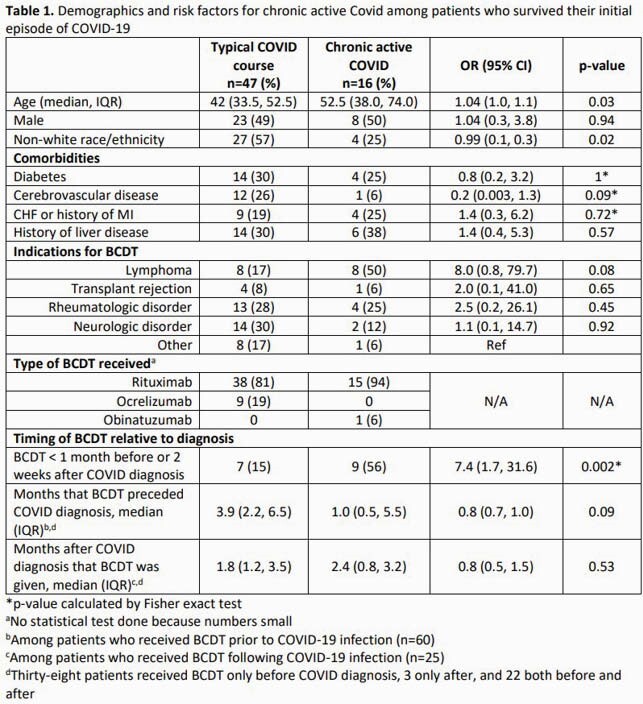

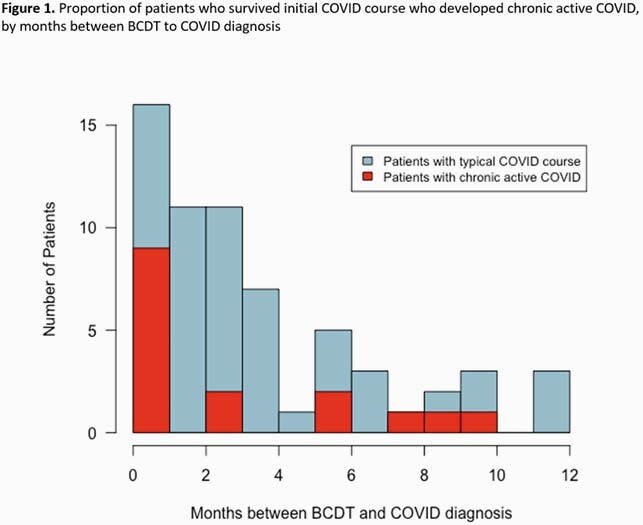

**Conclusion:**

We clinically observed COVID-19 infection lasting longer than the typical course and propose a definition for CAC. Incidence of CAC was highest among patients who received BCDT within 30 days before or 2 weeks after COVID-19 diagnosis. High suspicion for CAC is warranted among patients receiving these therapies. Additional study is needed to better define risk for CAC among varying immunosuppressed populations and determine whether COVID-specific treatments early in disease may benefit these patients.

**Disclosures:**

**Hannah Imlay, MD, MS**, **Gilead Sciences, Inc.** (Scientific Research Study Investigator)

